# #BrillantBeDient – Herausforderungen und Potenziale von Forschungsservices für dezentrale Forschung und Innovation im digitalen Raum

**DOI:** 10.1365/s40702-021-00721-3

**Published:** 2021-04-06

**Authors:** Anna M. Lux, Susanne Robra-Bissantz

**Affiliations:** Technische Universität Braunschweig, Institut für Wirtschaftsinformatik, Lehrstuhl für Informationsmanagement, Braunschweig, 38106 Deutschland

**Keywords:** Personennahe Dienstleistungen, Digitalisierung, Forschung, Innovation, Forschungsservice, Personal Services, Digitalization, Research, Innovation, Research Services

## Abstract

Digitale Services sind bereits Dreh- und Angelpunkt der Internetökonomie und bauen auf den Prinzipien von Interaktion, Zusammenarbeit und dem Wert individueller Unterstützung. Die Nutzung des Potenzials von digitalen Services ist in Praxisgebieten wie der Pflege, der Bildung und der Kinderbetreuung durch die Covid-19-Pandemie noch drängender geworden. Zur Schaffung von Innovationen in diesen Bereichen der personennahen Dienstleistungen, ist eine Zusammenarbeit von Wissenschaft und Praxis erforderlich. Doch die Mitwirkung in gängigen Kooperationsformen, wie z. B. Netzwerken, Technologieclustern oder Projekten, ist zunehmend von Dezentralität geprägt. Um im organisations- und standortübergreifenden Miteinander gemeinsame Ziele zu erreichen, bedarf es Unterstützungsleistungen. Der vorliegende Beitrag widmet sich den Herausforderungen und Potenzialen digitaler Forschungsservices dezentraler Forschung und Innovation. Dabei wird deutlich, dass die drei eingangs genannten Prinzipien nicht nur für die Internetökonomie von Bedeutung sind.

## Abstract (en)

Digital services are already the centerpiece of the Internet economy and are based on the principles of interaction, collaboration, and the consideration of individual values. Exploiting the potential of digital services in practice areas such as nursing, education, and child care, has become even more of a priority as a result of the Covid 19 pandemic. To bring out innovations in these areas of personal services, collaboration between science and practice is essential. But participation in common forms of cooperation, such as networks, technology clusters or projects, is increasingly characterized by decentralization. Support services are needed to achieve common goals in cooperation across organizations and locations. This contribution is dedicated to the challenges and potentials of digital research services for decentralized research and innovation. In this context, it becomes clear that the three principles mentioned at the beginning are not only relevant for the Internet economy.

## Zusammenarbeit: Überwindung von Krise und Raum

Was sich in einer Krise bewährt, verliert auch im Anschluss nicht an Bedeutung und bietet Chancen für eine nachhaltige Gestaltung vieler Bereiche der Gesellschaft. Die Covid-19-Pandemie führt zu **Veränderungen** unseres Miteinanders und lässt nicht-monetäre **Werte, Interaktion und Zusammenarbeit** in den Vordergrund rücken. Dies ließ sich in den vergangenen Monaten an vielen Situationen des Alltags festmachen, die geprägt waren von Eigeninitiative, Selbstorganisation, Kollegialität, Solidarität sowie einem neuen Zusammengehörigkeits- und Gemeinschaftsgefühl. Eine gesellschaftliche Tendenz, die bereits seit geraumer Zeit im analogen und digitalen Raum beobachtbar ist und durch die in der Krise rasante Digitalisierung beschleunigt wird: Die ubiquitäre Verbreitung digitaler Technologien in allen Bereichen unserer Gesellschaft führt zur Erosion tradierter Marktstrukturen und schafft Platz für neue Wertschöpfungsprozesse. Es eröffnen sich teils disruptive Chancen für neue Geschäftsfelder, Arbeitswelten und Geschäftsmodelle. Beziehungen zu Kund*innen und Geschäftspartner*innen werden neu verhandelt, Versorgungsketten umgestaltet und Arbeitsmodelle flexibilisiert. Im Vordergrund der Internetökonomie, die vor allem auf den eingangs genannten Veränderungen baut, stehen **digitale Services **(Robra-Bissantz et al. 2021).

Im Umfeld der produkt- oder industrienahen Dienstleistungen, sind durch die Digitalisierung bereits innovative und zum Teil hochgradig individualisierte Dienstleistungen entstanden. Während diese Angebote jedoch primär auf Informationen, Automatisierung und ökonomischer Motivation fußen, liegt das Innovationspotenzial neuer Märkte in einer Ausrichtung auf den Menschen (Lattemann et al. 2020). Es sind die **personennahen Dienstleistungen**, die über alle Branchen hinweg Bedeutung erlangen und zu einer Neuformation des Marktes führen (Robra-Bissantz et al. 2021). Wie die Gestaltung digitaler Services in Praxisgebieten, wie z. B. Bildung, Gastronomie, Kinderbetreuung und Pflege helfen kann, wertvolle Unterstützung für Individuen zu schaffen, ist in Corona-Zeiten noch drängender geworden und rückt in das Interesse der Wissenschaft und der Wirtschaft.

Innovative Dienste, wie sie aus den personennahen Dienstleistungen abgeleitet werden können, werden auch für** Forschung und Innovation **wichtiger (Lux und Robra-Bissantz 2020). Überall dort, wo etwas Neues oder Innovatives geschaffen werden soll – ob in Großprojekten von Konzernen, in Innovationsabteilungen von Unternehmen, privaten Forschungsinstituten oder öffentlichen Hochschulen – bildet Interdisziplinarität die Basis. Sie spiegelt sich in Netzwerken, Technologieclustern, Hubs, Communities und Verbundprojekten wider und stellt einen internationalen Trend dar (Ledford 2015). Die Mitwirkung in diesen Kooperationsformen erfolgt organisations- und oft standortübergreifend, erfordert eine hohe Interaktion und wird meist koordinierend begleitet. Doch wie werden die gemeinsamen Ziele der Zusammenarbeit erreicht? Vor dem Hintergrund der Covid-Pandemie, flexiblen Arbeitsweisen und einer hohen Dezentralität in Forschung und Innovation braucht es **digitale Forschungsservices.**

## Von der Ubiquität personennaher Dienstleistungen

### Der Mensch als Antwort

Der Begriff der personennahen Dienstleistungen (PDL) ist bislang nicht eindeutig definiert. Im Allgemeinen werden dabei zunächst solche Dienstleistungen verstanden, die *an* einer Person durchgeführt werden, wie dies in der Medizin, Pflege, Kinderbetreuung oder Kosmetik der Fall ist. Weitergehend auch Dienstleistungen *für* die private Person, z. B. in Form von handwerklichen Leistungen, Beratung oder gastronomischen Services (Robra-Bissantz et al. 2021). Vollständig erfassen lassen sich PDL aber erst mit folgendem Verständnis: *PDL* schließen *alle direkt an der einzelnen Person und ihren Bedürfnissen ausgerichteten Angebote ein, die in ihrer Kernausprägung Individuen dabei helfen, für sich Werte zu schaffen oder Probleme zu lösen* (Lattemann et al. 2019). Die individuelle Wertschaffung erfordert eine hohe **Interaktion **und **Zusammenarbeit **mit dem Individuum, aber auch mit weiteren Dienstleistern, die zur Lösung beitragen (vgl. Grönroos 2011). Dabei sind PDL nicht nur als Angebote für eine Privatperson zu verstehen, sondern adressieren ebenso Aktionen im B2B-Geschäft . Letztlich prägt auch die zwischen Unternehmen erbrachte Leistung direkt oder indirekt einen Nutzen für einen Menschen aus, z. B. in dessen beruflichem Umfeld durch ein Update der Dokumentationssoftware und dadurch zusätzlich gewonnener Funktionen (Robra-Bissantz et al. 2021).

Gesellschaftliche Trends, wie sich verändernde Lebensstile, Konsummuster und Arbeitsweisen führen zu neuen Bedarfen an individuellen Unterstützungsleistungen im privaten und beruflichen Umfeld (Kleinschmidt et al. 2016) und lassen die Nachfrage nach PDL stetig steigen. Der Stellenwert PDL zeigte sich eindrucksvoll in der Corona-Krise, wo Leistungen zur Befriedigung menschlicher Bedürfnisse eine hohe Aufwertung erfuhren (z. B. Becka et al. 2020). Gleichzeitig wurde die Dringlichkeit spürbar, die Chancen des Einsatzes von Technologien im Bereich der PDL besser nutzbar zu machen (Lattemann et al. 2020). Eine Aufgabe, an der seit einigen Jahren in diversen Projekten geforscht wird, denn durch die Digitalisierung kann der zugrundeliegende Interaktionsraum PDL fundamental erweitert werden.

### Angekommen in der neuen Internetökonomie

Einhergehend mit dem Vormarsch der Digitalisierung lässt sich beobachten, wie langjährig erfolgreiche Unternehmen in ihrer Marktposition durch unbekannte Akteur*innen zurückgedrängt werden. Grundlage disruptiver Innovationen, wie sie bei Netflix, Spotify, Airbnb, Uber oder Amazon beobachtbar sind, ist die Erkenntnis, dass ein produktzentriertes Denken keinen Erfolg mehr verspricht (Robra-Bissantz et al. 2021). In der neuen Internetökonomie sind digitale Services innerhalb einfacher Systemlösungen die Basis der Wertschöpfung. Dreh- und Angelpunkt der unternehmerischen Ausrichtung ist der individuelle **Wert** des (digitalen) Angebots für Nutzer*innen. Um diesen Wert zu erreichen, braucht es die Zusammenführung von Kompetenzen über eine gleichberechtigte **Zusammenarbeit** auf verschiedenen Ebenen: zwischen Kund*innen, zwischen Organisationen und Kund*innen und zwischen Organisationen (Lusch und Nambisan 2015). Wo Personen mit ihren menschlichen Bedürfnissen im Vordergrund stehen, nimmt auch die Art und Weise, *wie* miteinander in Kontakt getreten und agiert wird, die **Interaktion**, Einfluss auf das Gelingen (Geiger et al. 2020).

Insbesondere für industrielle Organisationen ist dieser beobachtbare Paradigmenwechsel des Marktes noch schwer zu realisieren, der sich – trotz möglicher Differenzierungsgrade – als Kernaussage zusammenfassen lässt: ***Sämtliche Dienstleistungen sind personennahe Dienstleistungen ***(vgl. Robra-Bissantz et al. 2021).

### Vom Eigenbedarf der Forschung

Die Nachfrage nach bedürfnisorientierten, innovativen und digitalen Angeboten wächst zunehmend auch im Praxisgebiet der Wissenschaft – man könnte auch sagen, die Nachfrage nach personennahen Dienstleistungen nimmt zu. Mit Nachdruck und großer Dringlichkeit zeigte sich dies im Bereich der *Lehre und Verwaltung*, als es im Frühjahr 2020 galt, Hochschulen im massiv eingeschränkten Betrieb aufrechtzuerhalten. Mit großem Aktionismus und großer Experimentierfreudigkeit gelang es in kurzer Zeit, eine digitale Lehre anzubieten. Im Bereich der *Forschung* zeichnet sich an Hochschulen bereits seit einigen Jahren ein steigender Bedarf an Unterstützung individueller oder kollaborativer Forschungsprozesse ab. Einflussfaktoren hierfür sind organisatorische und strukturelle Veränderungen, sinkende Grundmittel bei gleichzeitig steigenden Drittmitteln sowie eine Zunahme inter- und transdisziplinärer Aktivitäten in Form von überregionalen und internationalen Kooperationen, z. B. Arbeitsgruppen, Labs und Förderprojekten (Lux und Robra-Bissantz 2020).

Unterstützende Dienstleistungen werden teils durch neue berufliche Rollen abgebildet, die das akademische Personal ergänzen (z. B. Heuer 2018; Schneijderberg und Teichler 2013). Koordinator*innen, Forschungsmanager*innen oder Geschäftsführer*innen entlasten Wissenschaftler*innen in wissenschaftsperipheren Verpflichtungen und unterstützen sie so in ihrer originären Aufgabe – der Forschung. Auch in dezentraler Forschung, wie sie z. B. durch öffentlich geförderte Projekte entsteht, erbringen koordinierende Stellen Services für Forschungsverbünde, indem sie die organisatorische Basis verantworten und Arbeiten zwischen den Fachdisziplinen abstimmen. In Projekten mit transdisziplinärem Charakter befördern sie, über den Einbezug von Anwender*innen aus Unternehmen oder gesellschaftlichen Gruppen, direkt oder indirekt Innovation (Lux und Robra-Bissantz 2020).

In der Frage, wie sich der steigende Bedarf an Forschungsservices durch geeignete (personennahe) Dienstleistungen abbilden lässt, fehlt es bislang noch an Gestaltungsempfehlungen. Ebenso steckt die Erkundung digitaler Services hier noch in den Anfängen und bedarf der intensiven, praxisorientierten Forschung. Nachfolgend wird zu eben dieser Betrachtung ein Beispiel herangezogen.

## Dezentrale Forschung und Innovation begleiten – ein Praxisbeispiel

### Begleitforschung personennaher Dienstleistungen (BeDien)

Seit Ende 2018 wird vom Bundesministerium für Bildung und Forschung (BMBF) das Metaprojekt „Begleitforschung Personennaher Dienstleistungen“ (BeDien) gefördert. Innerhalb der dazugehörigen Bekanntmachung untersuchen acht transdisziplinäre Verbundprojekte das Potenzial von Technologien und digitalen Services für personennahe Dienstleistungen. Mehr als 50 Beteiligte aus Wissenschaft und Wirtschaft sind deutschlandweit involviert und fokussieren Anwendungsfelder wie z. B. Wohnen, Handwerk, Bildung und soziale Dienste. Der zentrale Auftrag von BeDien liegt darin, die Verbundprojekte in Forschung und Innovation zu unterstützen und die Anschlussfähigkeit des Themenfeldes personennaher Dienstleistungen (PDL) im wissenschaftlichen, praktischen und gesellschaftlichen Diskurs zu ermöglichen. Darüber hinaus geht das Metaprojekt auch eigenen Forschungsarbeiten nach.

Dienstleistungen, die BeDien erbringt, richten sich vorwiegend an alle beteiligten Projektpartner*innen der in der Bekanntmachung geförderten Verbundprojekte. Entlang der Erkenntnisse aktueller Dienstleistungsforschung, wird der Nutzen der Services mit den Projekten gemeinsam realisiert. Darüber hinaus richten sich die Angebote ebenso an den Projektträger, Stakeholder der Projekte (z. B. Verbände, Kammern, assoziierte Partnereinrichtungen), die Wissenschaft und die Wirtschaft im Allgemeinen sowie Bürger*innen. Das Konzept umfasst mehrere Präsenzbausteine, ergänzt durch vielfältige digitale Formate auf der Projektwebseite (www.bedien.org) und in den sozialen Medien. Um die bisherigen Angebote anzupassen oder weiterzuentwickeln, war zur Mitte der Projektlaufzeit eine Zwischenevaluation vorgesehen. Da diese mit dem pandemiebedingten Wegfall von Präsenzangeboten zusammenfiel, lag es nahe, die Ergebnisse zur Reflektion digitaler Unterstützungsmöglichkeiten heranzuziehen. Hierfür sollten die bisher qualitativ und explorativ zusammengestellten Aufgabenbereiche auf Vollständigkeit geprüft und hinsichtlich ihrer Relevanz eingeordnet werden. Des Weiteren umfasste die Umfrage auch spezifische Dienstleistungen von BeDien und ihre Bewertung hinsichtlich Wahrnehmung, Nutzung und Mehrwert.

Nachfolgend werden die Teilergebnisse der Umfrage vorgestellt, die sich auf die Aufgabenbereiche beziehen.

### Bereiche der Serviceentwicklung – Ergebnisse einer Zwischenevaluation

Die Umfrage wurde im Herbst 2020 online durchgeführt. Es nahmen 20 Personen teil, davon 17 als Vertreter*innen der Verbundprojekte und 3 als Vertreter*innen des Projektträgers. Gemäß der primären **Rolle im Projekt **teilten sich die Teilnehmenden auf in Partner*innen aus der Wissenschaft (40 %), Koordinator*innen (35 %), Projektbetreuer*innen (15 %) und Anwendungs- bzw. technische Partner*innen (je 5 %). **Erfahrung mit Begleitforschung** vor BeDien brachten bereits 45 % der Teilnehmenden mit. In der **Bewertung verschiedener Aufgabenbereiche einer Begleitforschung** (Tab. 1) hinsichtlich ihrer Wichtigkeit im Allgemeinen (Frage 5), gaben die Teilnehmenden an, dass die „projektübergreifende Zusammenführung von Erkenntnissen für die Praxis“ „sehr wichtig“ oder „wichtig“ sei (85 %). An zweiter Stelle wurden „Angebote zur projektübergreifenden Vernetzung“ (85 %) genannt, gefolgt von „projektübergreifende Zusammenführung wissenschaftlicher Erkenntnisse“ (75 %).

**Tab. 1:** Auszug aus der Zwischenevaluation. Ergebnisse zur Frage „Wie wichtig sind für Sie die nachfolgenden Aufgabenbereiche eines wissenschaftlichen Begleitforschungsprojektes im Allgemeinen?“

	Sehr Wichtig	Wichtig	Normal	Eher Unwichtig	Unwichtig	K. A.	Insgesamt	Gewichteter Mittelwert
Organisation allgemeiner Weiterbildungsangebote	10,00 % 2	20,00 % 4	45,00 % 9	10,00 % 2	15,00 % 3	0,00 % 0	20	3,00
Organisation projektspezifischer Weiterbildungsangebote	35,00 % 7	45,00 % 9	20,00 % 4	0,00 % 0	0,00 % 0	0,00 % 0	20	1,85
Angebote zur projektübergreifenden Vernetzung	40,00 % 8	45,00 % 9	15,00 % 3	0,00 % 0	0,00 % 0	0,00 % 0	20	1,75
Informationsbereitstellung und -verteilung	35,00 % 7	30,00 % 6	35,00 % 7	0,00 % 0	0,00 % 0	0,00 % 0	20	2,00
Projektübergreifende Öffentlichkeitsarbeit	31,58 % 6	31,58 % 6	31,58 % 6	5,26 % 1	0,00 % 0	0,00 % 0	19	2,11
Projektübergreifende (wissenschaftliche) Öffentlichkeitsarbeit	30,00 % 6	35,00 % 7	30,00 % 6	5,00 % 1	0,00 % 0	0,00 % 0	20	2,10
Projektübergreifende Zusammenführung wissenschaftlicher Erkenntnisse	50,00 % 10	25,00 % 5	25,00 % 5	0,00 % 0	0,00 % 0	0,00 % 0	20	1,75
Projektübergreifende Zusammenführung von Erkenntnissen für die Praxis	60,00 % 12	25,00 % 5	15,00 % 3	0,00 % 0	0,00 % 0	0,00 % 0	20	1,55
Durchführung ergänzender Forschungsarbeiten im Themengebiet	15,00 % 3	35,00 % 7	30,00 % 6	10,00 % 2	0,00 % 0	10,00 % 2	20	2,39

Darauf aufbauend wurden die Teilnehmenden gebeten, jene **Aufgabenbereiche** auszuwählen, die den größten Wert darstellen *für ihr Projekt, für sie selbst und für ihre berufliche Entwicklung*; eine Mehrfachnennung war möglich. Aus *Projektsicht* ergibt sich der größte Mehrwert laut Umfrage durch „Angebote zur projektübergreifenden Vernetzung“ (40 %), „Organisation projektspezifischer Weiterbildungsangebote“ (35 %) und „projektübergreifende Zusammenführung von Erkenntnissen für die Praxis“ (35 %). Für sich *persönlich* sehen die Teilnehmenden den größten Wert noch deutlicher in „Angebote zur projektübergreifenden Vernetzung“ (60 %). Ebenso werden auch die „Organisation projektspezifischer Weiterbildungsangebote“ (45 %) und die „projektübergreifende Zusammenführung wissenschaftlicher Erkenntnisse“ (30 %) von den Teilnehmenden als besonders relevant für sie selbst bewertet. Im Hinblick auf die berufliche Entwicklung fallen die Antworten etwas differenzierter aus. Hier wird die „Organisation projektspezifischer Weiterbildungsangebote“ am häufigsten ausgewählt (40 %), gefolgt von „Angebote zur projektübergreifenden Vernetzung“ (35 %) und „Projektübergreifende (wissenschaftliche) Veröffentlichungen“ (25 %).

Als besonders relevante Aufgabenbereiche lassen sich festhalten:

A. Projektübergreifende Zusammenführung von Erkenntnissen für die Praxis
B. Projektübergreifende Zusammenführung wissenschaftlicher Erkenntnisse
C. Projektübergreifende Vernetzung
D. Organisation projektspezifischer Weiterbildungsangebote

Die Aufgabenbereiche A und B beinhalten neben der Zusammenführung auch die Verbreitung der Erkenntnisse und lassen sich in diesem Verständnis auch als Wissenschaftskommunikation zusammenfassen. Dabei hat A eine deutliche Ausprägung in die Praxis zum Bereich „Innovation“, während B die „wissenschaftliche Dissemination“ fokussiert. Im nächsten Abschnitt werden durch einen Abgleich mit dem BeDien-Konzept Anregungen zu Services in den Aufgabenbereichen A bis D gegeben und aufgezeigt, wo aktuell ungenutzte Potenziale im digitalen Raum vorhanden sind.

## Service-Potenziale im digitalen Raum

### Wissenschaftskommunikation

Die Zusammenführung und Verbreitung gewonnener Erkenntnisse (Arbeitsbereiche A und B), spielt in vielen Formen der Zusammenarbeit eine Rolle. Dies mag unternehmensintern ebenso gelten, wie in einem wissenschaftlichen Fachbereich. Verbundstrukturen, in denen Praxis und Wissenschaft zu Forschungszwecken kooperieren, adressieren durch eine differente Aufbereitung und Kommunikation beide Bereiche. In öffentlich geförderten Zusammenschlüssen wird zudem noch ein größerer Wert auf die Darstellung der Forschungs- und Innovationsergebnisse für die breite Öffentlichkeit (Bürger*innen) gelegt. Im Sinne der Nachhaltigkeit ist zudem eine Offenlegung und Aufbereitung für die Praxis und ggf. auch die Politik von Interesse.

**Abb. 1:**
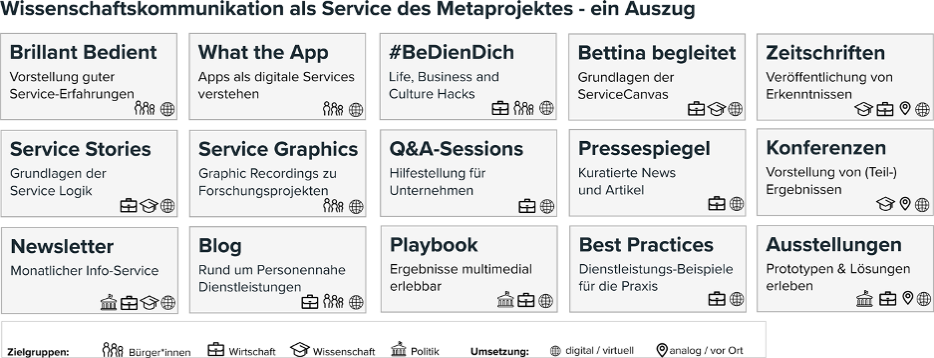
Darstellung von BeDien-Services in der Wissenschaftskommunikation

Im Gesamtkonzept von BeDien sind nahezu alle Aktivitäten darauf ausgerichtet in die Wissenschaftskommunikation einzuzahlen. Hier existieren im digitalen Raum bereits einige von BeDien entworfene und mit den Projekten weiterentwickelte Formate, die jeweils für unterschiedliche Zielgruppen (Bürger, Wirtschaft, Wissenschaft, Politik) gestaltet, aber gleichwohl für alle Interessierten, einschließlich der Projekt-Beteiligten zugänglich sind (vgl. Abb. 1).

So werden Erkenntnisse aus der Dienstleistungsforschung, die zur digitalen Transformation des eigenen (unternehmerischen) Angebotes herangezogen werden können, schrittweise in Erklärvideos („Bettina begleitet“) und Beitragsreihen („Susns Service Stories“) auf der Projektwebseite veröffentlicht (Bedien.org 2019). Positive Erfahrungen aus Alltags-Services werden als Podcast oder animiertes Video in „BrillantBeDient“ vorgestellt. So ergänzte BeDien z. B. als Reaktion auf die Corona-Krise Mitte 2020 die Webpräsenz um „Life, Business and Culture Hacks“. Unter dem Hashtag #BeDienDich wurden nützliche Tipps und Tricks für verschiedene Lebenssituationen angeboten, um die allgemeine Öffentlichkeit, aber auch speziell Einzelunternehmer*innen und Künstler*innen zu unterstützen (Bedien.org 2020). Ein frei zugänglicher Newsletter, regelmäßige Beiträge zu Neuigkeiten aus den personennahen Dienstleistungen und Aktivitäten auf verschiedenen Kanälen sozialer Medien vervollständigen die digitalen Services der allgemeinen Wissenschaftskommunikation.

Zur Dissemination wissenschaftlicher Ergebnisse stehen mittlerweile immer mehr OpenAccess-Beiträge bereit. Konferenzen und Tagungen, die in Präsenz geplant waren, wurden auf online-Formate umgestellt, sodass hinsichtlich der Verbreitung von Forschungsergebnissen keine Nachteile entstanden sind. Innovation wird durch Online-Methodenschulungen und die Aufbereitung der Endergebnisse für die Wirtschaft in einem Playbook umgesetzt, welches digital verfügbar sein wird und eine intermediale Verknüpfung zu Online-Content vorsieht.

Insgesamt lässt sich festhalten, dass für die Aufgabenfelder A und B bereits viele Möglichkeiten im digitalen Raum vorhanden sind, die für Forschungsservices genutzt und die miteinander verknüpft werden können.

### Vernetzung

Um der Vernetzung (Arbeitsbereich C) verschiedener Gruppen, wie sie im Beispiel durch die Projektverbünde dargestellt sind, nachzukommen, ist es nicht mit der Berücksichtigung von Kaffeepausen oder einem Get-Together am Buffet getan. Es entsteht erst dann ein Service, wenn *relevante* Begegnungen ermöglicht und befördert werden. Im besten Fall sind dies solche, die langfristig in das eigene Netzwerk übergehen. Hierzu ist es notwendig, die Interessen und Themenschnittpunkte des Anderen zu kennen und dessen Positionen, Kompetenzen und Expertisen zu verstehen. Die Gestaltung solcher Interaktionsräume und die moderative Zusammenführung von Menschen in wertvollen Begegnungen, stellt einen großen Service dar.

**Abb. 2:**
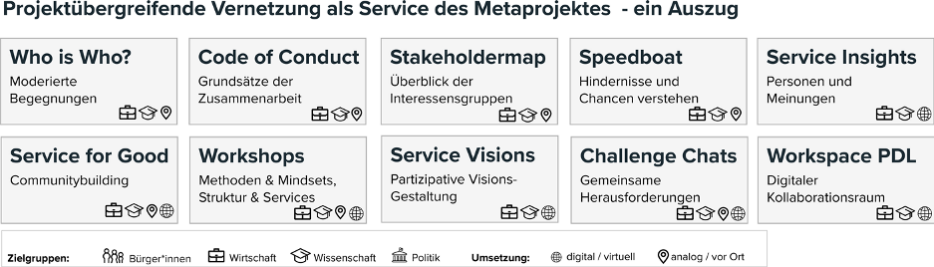
Darstellung von BeDien-Services zur projektübergreifenden Vernetzung

Auf eben solche Angebote hat das BeDien-Projekt in der ersten Projekthälfte einen besonderen Akzent gelegt und damit bei den Projekten und auch dem Projektträger eine sehr positive Resonanz erzielt. Abb. 2 fasst im Auszug einige Services des Metaprojektes zusammen.

Bereits in der Kick-Off-Veranstaltung standen Diskussionsrunden mit wechselnder Besetzung der Gruppenmitglieder, Speed-Dating und World-Cafés im Vordergrund. Ergebnisse, wie z. B. eine Stakeholder-Map oder ein gemeinsamer Code-of-Conduct, wurden unter Mitwirkung wirklich aller Teilnehmenden erreicht. Auch in Folgeveranstaltungen, die als Präsenzbausteine im physischen Raum stattfanden (Workshops, Weiterbildungen oder Netzwerktreffen) wurden Interaktionen zwischen Beteiligter verschiedener Projekte stets durch Moderationstechniken und Methoden befördert, die gleiche Interessen und Themenschnittpunkte herausarbeiten ließen. Dabei wurde auch ein besonderes Augenmerk gelegt auf die Gestaltung des physischen Raumes und der eingesetzten Hilfsmittel. Ergänzend hierzu entstanden als digitaler Service die „Service-Insights“-Interviews zur Vorstellung einzelner Projektpartner*innen. Nachdem physische Formate im letzten Jahr nicht möglich waren, wurde eine projektübergreifende Plattform zur Zusammenarbeit eingerichtet, die kurze Kommunikationswege bietet und den Austausch in Foren (wie z. B. „Suche“, „Biete“ und „aktuelle Angebote“) erlaubt. Darüber hinaus wurden offene, digitale Kaffeepausen angeboten.

Das vergangene Jahr hat gezeigt, dass Veranstaltungen mit vorwiegend informativem oder unterhaltendem Charakter, wie z. B. Besprechungen, Vorträge und Messen, im virtuellen Raum gut funktionieren können. Hinsichtlich von Networking, Interaktion und Zusammenarbeit in größeren Gruppen, liegen die Stärken jedoch bisher in Präsenzveranstaltungen. Ihr Ersatz, d. h. die Gestaltung **synchroner **Interaktionsformate im digitalen Raum, ist noch Neuland. Der Funktionsumfang von Videokonferenzsystemen und ergänzenden Tools nimmt zwar zu, wie z. B. Social-Walls, Voting-Buttons und Tag-Clouds zeigen. Doch braucht es noch die Entwicklung und Erprobung neuer Konzepte zur Gestaltung interaktiver Online-Formate, um projektübergreifende Vernetzungsmöglichkeiten auch im digitalen Raum zu wertvollen Angeboten zu machen. Hier liegt noch viel Potenzial für Forschungsservices.

### Weiterbildung

In jeglicher Form der Zusammenarbeit, können Weiterbildungen zum gemeinsamen Fortschritt beitragen. Zwar sind Partner*innen in Konsortien, Netzwerken, Verbünden oder Projektteams häufig in Hinblick auf die Gesamtzielsetzung bereits mit den erforderlichen Kompetenzen ausgestattet, trotzdem kann sich im Verlauf der Bedarf nach externer Expertise auftun. Dieser kann vielfältig begründet sein: Impulse können benötigt werden, um Blickwinkel zu eröffnen oder Anregungen für eine andere Ausrichtung zu geben. Spezifische Beratung mag notwendig sein, um Hürden zu beseitigen oder Sicherheit zu erlangen. Oft mangelt es auch an neuen Methoden und Strukturen. Zudem fordern die Digitalisierung und Individualisierung von Leistungen heterogene und spezialisierte Fähigkeiten, die zusätzlich erworben werden müssen.

**Abb. 3:**
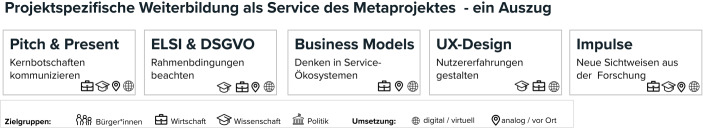
Darstellung von BeDien-Services in der projektspezifischen Weiterbildung

Das BeDien-Projekt unterstützt solche Bedarfe, indem es Wissen zu Methoden, Formaten und Strukturen vermittelt (vgl. Abb. 3). Durch die eigene Forschungsarbeit kann fachliches Know-how entweder selbst zur Verfügung gestellt, aus dem Netzwerk der Stakeholder vermittelt oder entsprechend der Anforderungen der Anfrage durch passende externe Dienstleister hinzugeholt werden. So gibt BeDien z. B. Einblicke in Forschungsmethoden der Praxis und trägt Impulse aus der Dienstleistungsforschung in die Projekte. Zur gezielten Stärkung der Kompetenzen der Projekte in Hinblick auf die Projektphase, werden ausgewählte Vorträge oder Workshops angeboten. Während des Projektbeginns waren dies z. B. Themen wie Datenschutz und Webpräsenz. Zur Mitte der Förderzeit standen dagegen Geschäftsmodellentwicklung, UX-Design und Serviceinnovation im Vordergrund. Die Umsetzung in digitale Formate erfolgte im vergangenen Jahr durch wiederkehrende Q&A-Sessions mit Expert*innen, live-Übertragungen von Impulsvorträgen und digitalen Weiterbildungen durch Externe.

Es lässt sich feststellen, dass die ortsunabhängige Umsetzung von Weiterbildungsangeboten im digitalen Raum Vorteile bietet, insbesondere bei dezentraler Forschung. Durch die im Home-Office gestiegene Akzeptanz digitaler Besprechungen, die zunehmende Sicherheit im Umgang mit Konferenzsystemen und das steigende Angebot technischer Möglichkeiten sind Angebote hier gut umsetzbar. Den Herausforderungen in der digitalen Wissensvermittlung, wie z. B. die Aufrechterhaltung der Aufmerksamkeit, die Bestimmung der optimalen Gruppengröße, Fragen- und Feedbackrunden oder die Gestaltung von Lerneinheiten, steht ein wachsender Erfahrungsschatz gegenüber. Berücksichtigt man Empfehlungen zur Gestaltung und Hinweise zur Umsetzung von Impulsvorträgen, Webinaren oder Trainings, können wertvolle digitale Forschungsservices im Arbeitsbereich D entstehen. Worin noch Potenzial liegt und wo es noch an Lösungen fehlt, ist die Gestaltung der synchronen Interaktion zwischen den Teilnehmenden und deren Zusammenarbeit. Aspekte, die im physischen Raum in den Pausen, in Gruppenarbeiten oder als Teil des Veranstaltungskonzeptes (z. B. Warm-Up) berücksichtigt würden.

## Brillant bedient – ein Dreisatz im digitalen Raum

Aus den vorherigen Betrachtungen lässt sich zeigen, dass Forschungsservices im digitalen Raum aktuell noch dort an ihre Grenzen stoßen, wo synchrone Interaktionen im Vordergrund der Zusammenarbeit stehen. Doch gerade diese spielen in der zunehmenden Dezentralität von Forschung und Innovation eine primäre Rolle. Die Mitwirkung in standortübergreifenden Kooperationsformen ist nicht immer in-Person möglich. Die Corona-Krise hat die Vorteile von Online-Treffen deutlich aufgezeigt und dem Trend der interdisziplinären Zusammenarbeit Wege für künftige Formen des Begegnens gefestigt. Kosten und Aufwand von Reisen werden künftig kritisch abgewogen werden müssen, gegenüber einem Zusammenkommen im virtuellen Raum.

Auch in vielen anderen Bereichen unserer Gesellschaft hat die weltweite Pandemie zu einer Beschleunigung bereits vorhandener Tendenzen geführt, wie sie in flexiblen Arbeitsmodellen, digitaler Verwaltung und virtuellen Sprechstunden deutlich werden (Schüür-Langkau 2020). Die Umsetzung birgt teils noch Startschwierigkeiten, doch kann in der technischen Basis – Digitalisierung und Vernetzung – grundsätzlich keine Herausforderung mehr gesehen werden. Vielmehr stellt sich die Frage, wie Technik eingesetzt wird, um die Gestaltung des Miteinander sinnvoll und reibungslos umzusetzen (Jandrić et al. 2018). Hier zeigt sich, in der Corona-Krise im physischen Raum und in der Krise der digitalen Transformation, dass im Mittelpunkt aller Prozesse und Handlungen der Mensch stehen muss (vgl. Abschn. 2). Im Aufbau von Angeboten personennaher Dienstleistungen – physisch, digital oder digitalisiert – werden drei Kernelemente wichtig: Der **Wert **des Angebotes, der sich individuell und kontextabhängig entfaltet (Chandler und Vargo 2011); die **Interaktion **(Geiger et al. 2020), für die dafür notwendige Abstimmung und das Gelingen der **Zusammenarbeit **(z. B. Lusch und Nambisan 2015), in der gemeinsamen Gestaltung eines wertvollen Angebotes (vgl. Lattemann et al. 2020).

Diese Perspektive braucht es künftig in jeglichen Dienstleistungen, auch in digitalen Services für Forschung und Innovation, wie sie Betrachtungsgegenstand des Artikels sind. Hier gilt es noch zu lernen, die Berücksichtigung von Werten besser zu verstehen. Letztlich wirkt digitaler Service *wertvermittelnd* in den physischen Raum des Menschen: die Online-Bestellung, der Stromanbieterwechsel über die Vergleichsplattform, die Buchung einer Unterkunft. Die aufgeführten Herausforderungen in der Umsetzung digitaler Services zeigen sich vom Wert aus. So bieten z. B. die bisherigen Konzepte für digitale Interaktion noch nicht den gleichen Wert, wie er in Präsenz entsteht. Vernetzung und Zusammenarbeit bedingen intensive Gespräche, den Austausch von Standpunkten, die Identifikation gemeinsamer Interessen und letztlich auch eine persönliche Einschätzung des Gegenübers. Mit steigender Erfahrung, neuen Konzepten und Arbeitsweisen für das Zusammenspiel von Mensch, Raum und Technik, können unter Berücksichtigung der drei Kernelemente – Wert, Interaktion, Zusammenarbeit – künftig viele brillante (Forschungs‑)Services entstehen.
